# Design, recruitment, and retention of African-American smokers in a pharmacokinetic study

**DOI:** 10.1186/1471-2288-10-6

**Published:** 2010-01-19

**Authors:** Babalola Faseru, Lisa S Cox, Carrie A Bronars, Isaac Opole, Gregory A Reed, Matthew S Mayo, Jasjit S Ahluwalia, Kolawole S Okuyemi

**Affiliations:** 1 Department of Preventive Medicine and Public Health, University of Kansas Medical Center, Kansas City, KS, USA; 2 Department of General and Geriatric Medicine, University of Kansas Medical Center, Kansas City, KS, USA; 3 Department of Pharmacology, Toxicology and Therapeutics, University of Kansas Medical Center, Kansas City, KS, USA; 4 Department of Biostatistics, University of Kansas Medical Center, Kansas City, KS, USA; 5 Department of Medicine, University of Minnesota Medical School, Minneapolis, MN, USA; 6 Department of Family Medicine and Community Health, University of Minnesota Medical School, Minneapolis, MN, USA; 7 Program in Health Disparities Research, University of Minnesota Medical School, Minneapolis, MN, USA

## Abstract

**Background:**

African-Americans remain underrepresented in clinical research despite experiencing a higher burden of disease compared to all other ethnic groups in the United States. The purpose of this article is to describe the study design and discuss strategies used to recruit and retain African-American smokers in a pharmacokinetic study.

**Methods:**

The parent study was designed to evaluate the differences in the steady-state concentrations of bupropion and its three principal metabolites between African-American menthol and non-menthol cigarette smokers. Study participation consisted of four visits at a General Clinical Research Center (GCRC) over six weeks. After meeting telephone eligibility requirements, phone-eligible participants underwent additional screening during the first two GCRC visits. The last two visits (pharmacokinetic study phase) required repeated blood draws using an intravenous catheter over the course of 12 hours.

**Results:**

Five hundred and fifteen African-American smokers completed telephone screening; 187 were phone-eligible and 92 were scheduled for the first GCRC visit. Of the 81 who attended the first visit, 48 individuals were enrolled in the pharmacokinetic study, and a total of 40 individuals completed the study (83% retention rate).

**Conclusions:**

Although recruitment of African-American smokers into a non-treatment, pharmacokinetic study poses challenges, retention is feasible. The results provide valuable information for investigators embarking on non-treatment laboratory-based studies among minority populations.

## Background

Although African-Americans experience a disproportionately high burden of disease [1,2], they remain underrepresented in clinical research [3-14]. Clinical research studies traditionally have included predominantly White samples and only a small percentage of minority participants. Therefore, findings of these studies have limited generalizability to African-Americans or other racial/ethnic minority populations [5,9,15-18]. Reasons for low levels of participation of African-Americans in research studies have included mistrust of the medical and research communities, lack of awareness of potential benefits of study programs, and barriers related to low economic status [12,19]. While some investigators are beginning to address these barriers [20-25], recruitment and retention of African-Americans in clinical research studies remain a critical challenge [26].

Tobacco-related morbidity and mortality is higher among African-Americans compared to other racial/ethnic groups in the United States [27]. However, African-Americans remain underrepresented in smoking cessation and tobacco research [28]. While about 80% of African-American smokers smoke menthol cigarettes [29-31], smoking menthol cigarettes is associated with decreased abstinence following bupropion treatment in African-American smokers [32,33]. The purpose of the parent study was to evaluate the differences in the steady-state concentrations of bupropion and its three principal metabolites between African-American menthol and non-menthol cigarette smokers. Understanding the effect of menthol on bupropion metabolism could inform the choice of smoking cessation medications to African-American smokers. This study is unique and presents recruitment and retention challenges for the following reasons: 1) unlike clinical trials, this study was laboratory-based and involved a non-treatment-seeking study population; 2) participants were neither required to be interested in quitting nor motivated to quit; 3) participants were not in need of clinical investigation or treatment for any physical illness that would motivate them to visit the hospital multiple times and stay in the hospital for long hours of investigation. This article describes the design, successful recruitment and retention of African-Americans in our pharmacokinetic study. We will discuss challenges and solutions regarding recruitment and retention issues in the study, which could provide useful information for investigators embarking on similar research among minority populations.

## Methods

The study protocol was reviewed and approved by the University of Kansas Medical Center Human Subjects Committee. This study was conducted within the General Clinical Research Center (GCRC) at the University of Kansas Medical Center.

### Study Design

**Figure 1:**
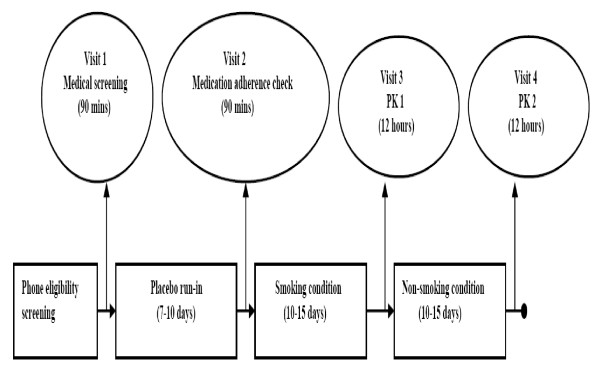
**Overview of the study design to evaluate the effect of menthol on the pharmacokinetics of bupropion and its metabolites**.

Figure 1 provides an overview of the study that included screening and four visits to the GCRC. The study was designed to provide information about possible interaction between menthol in mentholated cigarettes and bupropion (Zyban^®^, Glaxo SmithKline) by evaluating the effects of menthol in mentholated cigarettes on the pharmacokinetic (PK) profiles of bupropion and its three principal metabolites: hydroxybupropion, threohydrobupropion, and erythrohydrobupropion. The study aimed to recruit and enroll 20 African-American smokers of mentholated cigarettes (menthol smokers) matched 1:1 with 20 African-American smokers of non-mentholated cigarettes (non-menthol smokers) by gender, number of cigarettes per day smoked (CPD), and body mass index (BMI). Pharmacokinetic (PK) parameters of bupropion and its three principal metabolites were assessed at steady state under smoking and non-smoking conditions. During the smoking condition which lasted 10-15 days, subjects smoked their usual brand of cigarettes. This period was followed by a non-smoking condition lasting another 10-15 days. The PK parameters were then assessed between 1) menthol and non-menthol smokers, and 2) smoking and non-smoking conditions.

### Preparatory Phase

#### Recruitment

Participants were recruited using clinic-based and community-based strategies. Clinic-based strategies included informing medical center staff about the study through flyers, broadcast emails, departmental meetings, and employee newsletters. Posters and flyers were distributed at a community health center that serves a predominantly African-American patient population. Invitation letters were sent to former research participants who gave written consent to be contacted in the future. Community strategies included paid advertisements in neighborhood newspapers, the major city paper, and on two radio stations with large African-American audiences. Research staff provided flyers to African-American business owners and religious organizations. Those interested in the study were asked to call the study office and speak with a study coordinator who assessed their eligibility for the study.

#### Eligibility criteria

Eligible individuals identified themselves as African-American or Black, aged 18 years or older. They smoked at least 10 cigarettes per day and had a BMI between 18 and 45 kg/m^2^. Additionally, each participant must have smoked either mentholated or non-mentholated cigarettes exclusively for the past year. Consistent with contraindications for bupropion use, exclusion criteria included predisposition to seizures, a diagnosis of bulimia or anorexia nervosa in the past year, an unstable medical or psychiatric illness, alcohol dependency within the last year, or a myocardial infarction in the last month. In addition, individuals who planned to move from the metropolitan area in the next month, or who had used other forms of tobacco in the past 30 days, or had used bupropion in the past 30 days were excluded from participation, as were those currently using any prescription or other medications contraindicated or with known interaction with bupropion, those reporting illicit drug use and women who were pregnant, breastfeeding, or contemplating pregnancy in the next month.

#### Eligibility Screening

Eligibility screening was multi-staged: telephone eligibility screening was followed by medical screening, and then, lastly, by screening for adverse events with medication and adherence to medication.

#### Telephone Screening

Study staff specifically informed callers that this was a "non-treatment study to find out how the body breaks down a medication called Zyban". Research staff verified individuals' willingness to adhere to study instructions. Interested individuals were screened on the phone according to the eligibility criteria described above. Those eligible were informed that medical screening would include an additional medical history, a physical examination and laboratory tests to rule out contraindications to bupropion use. They were instructed to fast overnight prior to the medical screening visit. A written copy of these instructions was mailed to all individuals who had passed the telephone screening.

#### Medical Screening

Phone-eligible individuals came to the General Clinical Research Center (GCRC) after an overnight fast. The study staff discussed the details of the study with participants during the informed consent process. Individuals signed a written informed consent form. They provided a 20 ml blood sample for a complete blood count, a complete metabolic profile, and cotinine analysis. Women also gave a urine sample for a pregnancy test. The GCRC nurse took a medical history and examined the participants. Participants completed a baseline smoking history questionnaire and also completed a smoking topography using a CreSSmicro smoking topography device (Plowshare Technologies, Baltimore, MD). The study staff and the study physician reviewed the results of the laboratory tests, medical history and physical examination findings to determine eligibility. Eligible participants were given a sterile container for collecting urine beginning 24 hours prior to the next visit to verify their menthol status. They were also given a special cigarette carrying container (Smartpak) to use during the study.

#### Reporting adverse events and verifying medication adherence

Medically eligible participants were given a 7-10 day supply of placebo in a container with a Medication Event Monitoring System (MEMS) cap, an adherence verification device (AARDEX, Union City, CA). On their second visit, participants were asked about adverse events using the Common Terminology Criteria for Adverse Events, version 3.0 (National Cancer Institute). Unused study medication was collected and counted, and data were downloaded from the MEMS cap. Individuals who did not tolerate the placebo medication or used less than 75% of the prescribed dose were excluded from continued participation. Such individuals were excluded with the rationale that they were unlikely to take the active medication as prescribed during the entire study. Participants were enrolled in the pharmacokinetics phase of the study after they passed the medical adherence eligibility screening (Visit 2), and were scheduled for Visit 3. The participants continued to use the MEMS cap for the entire study to monitor bupropion use. The study staff instructed participants to bring their MEMS cap and all medications to each GCRC visit.

### Pharmacokinetic Study Phase

#### Procedure overview

After a seven-day placebo run-in period and an initial three-day dosing period of 150 mg/day, participants were given 300 mg/day (150 mg 2×/day) sustained-release bupropion for 20-25 days. Participants were asked to smoke their usual brand of cigarettes *ad lib *for the first 10-15 days (smoking condition) and to quit smoking for the remaining 10-15 days of the study (non-smoking condition). Blood samples were drawn for pharmacokinetics (PK) analysis on two occasions, 10-15 days after the commencement of bupropion while participants were still smoking (PK 1), and again at days 20-25 (PK 2) when they were required not to smoke (non-smoking condition). The blood samples at Visit 3 (PK 1) provided PK parameters when participants were exposed to both bupropion and menthol in cigarettes. Samples at Visit 4 (PK 2) provided PK parameters when participants were exposed only to bupropion but not to menthol. At each PK visit, approximately 10 ml of blood specimens for PK were taken through an intravenous line inserted into the participant's arm prior to ingestion of 150 mg bupropion-SR and at 1, 2, 2.5, 3, 3.5, 4, 5, 6, 8, and 12 hours after ingestion of the first dose of 150 mg bupropion-SR. Use of the second dose of bupropion for the day was delayed until after all blood draws were completed.

### Retention Strategies

#### Reminders

Once participants were scheduled for visits, reminder letters were mailed one week before each visit, and reminder telephone calls were placed two days and one day before each visit. The study staff provided information about the procedures that participants would undergo in the upcoming visit and answered questions. Participants who missed an appointment were contacted by telephone to reschedule the appointment, provided they were still within the study visit window. The research team met weekly to review recruitment and retention progress and to provide feedback to the study staff.

#### Incentives

Monetary compensation was discussed with participants during the informed consent process. Participants were given $50 Visa gift cards for each screening visit and $150 Visa gift cards for each pharmacokinetic study visit. Fifty dollar Visa gift cards were given to the participants for the use of Smartpak and MEMS devices. Additional $50 Visa gift cards were given to participants who were able to abstain from smoking during the non-smoking phase (verified with Nic check).

#### Data Analysis

This is a descriptive summary and, as such, we summarized categorical variables by frequencies and percentages and continuous variables by means and standard deviations using SAS statistical package, version 9.1.

## Results

**Figure 2:**
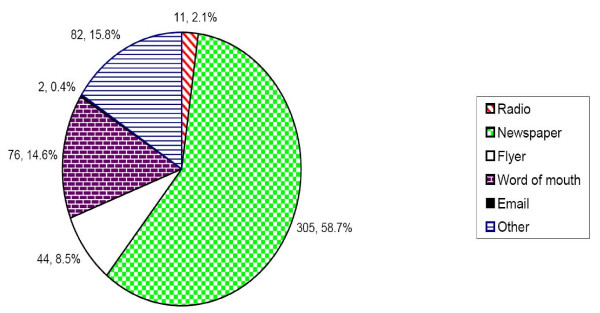
**Sources of information about study**. *The distribution represents 520 responses from 515 individuals screened on the phone.

**Figure 3:**
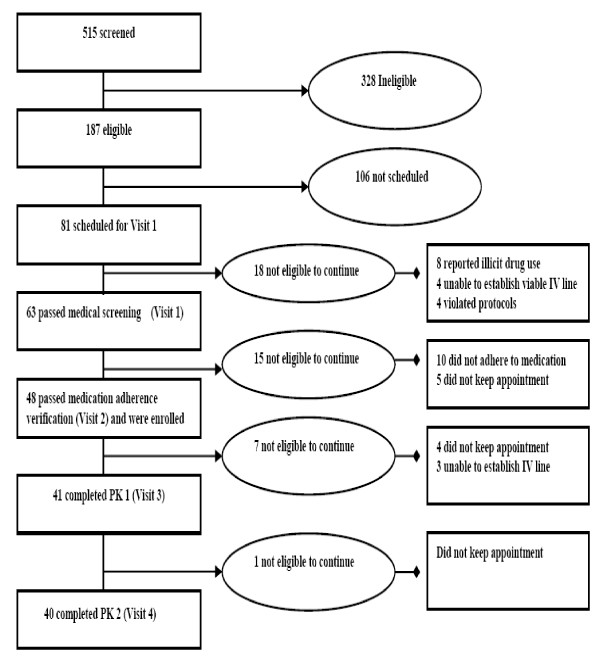
**Flow chart showing the recruitment and retention of participants in the pharmacokinetics study**.

Recruitment was conducted from February 2006 to February 2007, during which time a total of 515 African-American smokers were screened via telephone. Interested individuals learned about the study from newspapers (58.7%), word of mouth (14.6%), and flyers (8.5%) See Figure 2. Similarly, newspaper advertisement produced the highest yield of participants enrolled in the pharmacokinetic study. Of the 515 screened, 187 were phone-eligible (Figure 3). Some of the reasons for ineligibility during phone screening included: smoking fewer than 10 cigarettes per day; not smoking menthol or non-menthol cigarettes exclusively; smoking other tobacco products such as "Black and Mild" cigars; excessive drinking of alcohol; and inability to attend 12-hour appointments.

**Figure 4:**
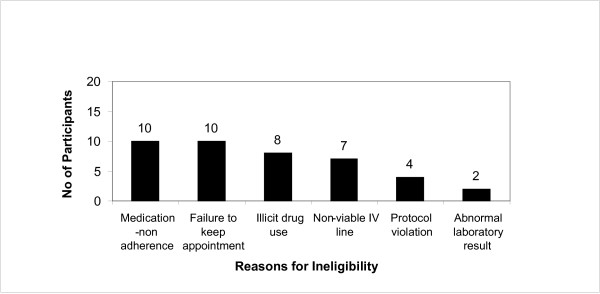
**Primary reasons for excluding participants from study after attending medical screening visit (n = 41)**.

In total, 92 smokers were scheduled for Visit 1, and 81 kept their appointments. Figure 3 presents an overview of participant retention, which shows 40 participants completing all study visits. Figure 4 provides a summary of the primary reasons for exclusion. Of the 81 smokers who attended Visit 1, 41 (50.6%) were excluded due to: medication non-adherence (12.3%); failure to keep appointments (12.3%); illicit drug use (9.9%); failure to establish IV lines (8.7%); protocol violations (4.9%); or abnormal test results (2.5%).

Table 1 presents the characteristics of the participants who were enrolled in the pharmacokinetic study (n = 48). Participants were approximately 51 (SD = 9.7) years old and smoked an average of 15.1 (SD = 4.6) cigarettes per day for a mean duration of 33 years. Fifty-eight percent were females. The average BMI was 29.9 (SD = 7.0), and the mean age of smoking initiation was 17.6 (SD = 4.4) years.

**Table 1:** Baseline characteristics of participants enrolled in the PK study (n = 48)

	All	Menthol (n = 29)	Non-Menthol (n = 19)	P value
Mean age (SD)	50.9 (9.7)	47.4 (9.3)	56.3 (7.9)	<0.001^1^
Gender, n (% female)	28 (58.3%)	16 (57.1%)	12 (42.9%)	0.77^2^
BMI*	29.9 (7.0)	30.5 (7.0)	29.0 (7.1)	0.48
Mean age of smoking initiation	17.6 (4.4)	18.0 (5.1)	16.9 (3.1)	0.34
Mean age of regular smoking	19.7 (5.0)	19.9 (5.9)	20.9 (4.5)	0.70
No. of years smoked	33.4 (10.1)	29.4 (9.4)	39.4 (7.8)	<0.001
Mean CPD at baseline	15.1 (4.6)	15.3 (5.2)	16.1 (3.7)	0.33^1^
FTND Score**	4.8 (1.7)	4.5 (1.6)	5.2 (1.8)	0.18

^1^Wilcoxon rank sum test; ^2^Fisher's exact test; all other p-values calculated with t -tests;

*Body Mass Index. **Fagerström Test of Nicotine Dependence.

## Discussion

This study demonstrated that African-American smokers can be successfully recruited to participate in an intensive GCRC-based non-treatment study. Given the intensity of participation required for the study, including 24-hour urine collection, the use of cigarette and pill monitoring devices, the number and duration of study visits, and multiple blood draws, achieving this recruitment level was considered a success. This outcome supports the feasibility of including African-Americans in laboratory-based research. Showing that African-Americans will volunteer to enroll in this type of research is important, given the persistent national problem of underrepresentation of African-Americans in clinical studies [10]. The recruitment goal of the study was to have 40 African-Americans complete the pharmacokinetic study. We screened 515 and identified 187 eligible African-American smokers. Of 81 individuals scheduled for the medical screening (Visit 1) at the GCRC, 49.4% completed the full PK study. It should be noted that it took approximately 12 months to meet this enrollment goal, a much longer time than is needed to recruit the same number of smokers in most smoking cessation studies [34-36]. This discrepancy is likely due to the intensive nature of the protocol as well as the location of the study. This study was located in an academic medical center and most of our participants heard about the study through newspaper advertisement. This is a departure from our previous study in a community health center serving a predominantly low-income African-American population. In that study, word of mouth yielded more participants [37]. Our advertisement strategies also played a role. Periodically, we reviewed progress with recruitment methods and invested more in newspaper advertisement, which proved to be more effective compared to other sources.

Multiple factors contributed to recruitment success. Our research team has a track record of successfully engaging African-Americans in research studies, and we applied this experience to our recruitment efforts [32,34,37-53]. We emphasized cultural competence and cultural sensitivity training within our research team. We involved the African-American community and health care providers through a Community Advisory Board in the planning and execution of our projects [34]. Our efforts in the community have progressively built trust, which is critical given that lack of trust is a major impediment to the participation of African-Americans in medical research studies [12]. We integrated recruitment tracking mechanisms to maximize retention of our participants. In this study, we achieved a relatively high retention rate of 83%. Of the 48 participants who met final eligibility criteria and enrolled in the pharmacokinetic study, 40 participants completed the study. This rate is similar to the retention rates of 84% we obtained in our previous clinical trials [52,54], but lower than 90%, reported in a more intensive long-term clinical trial involving African-Americans with renal insufficiency [55]. This implies that retention might be better in a treatment-seeking population than in a non-treatment-seeking population.

The recruitment of African-American non-menthol smokers into our study was particularly challenging. Only about 20% of African-American smokers smoke non-menthol cigarettes [29-32,56]. Because of the lower prevalence of African-American non-menthol smokers, we could not enroll eligible menthol smokers until we found non-menthol smokers who could be matched with menthol smokers. Consequently, we had a long waiting list of menthol smokers who, although eligible for the study, could not be enrolled due to lack of a non-menthol match. This sampling technique is prone to selection bias and may limit the generalization of our study findings. This limitation speaks to methodological challenges pertaining to research involving menthol and non-menthol African-American smokers.

The majority of the participants who were excluded from this study after the informed consent process either did not adhere to medication regimen or did not keep the scheduled appointment. We do not know if failure to attend visit during the abstinence phase was due to the fact that some of the smokers could not stop smoking during this phase. While the exclusion criteria were carefully chosen to remove individuals who seemed unlikely to comply with our protocol, this study also provided insight into issues that warrant further investigation, such as adherence to medication among minority populations, and factors that influence minority participants to not keep the study appointments. Illicit drug use (primarily marijuana) accounted for 19.5% of those who were excluded from study after consenting to participate. Although use of illicit drugs was a clear exclusion criterion, we administered this question in person because of its sensitive nature and the need to get more accurate information along with a certificate of confidentiality on their behalf from the federal government agency for their protection.

## Conclusions

Our study demonstrates the feasibility of recruiting African-American smokers to a pharmacokinetic study, and identifies unique challenges to recruitment and retention. These challenges include intense eligibility criteria with multiple levels of eligibility, rigorous protocol, and invasive procedures. Despite these, we have demonstrated that recruiting African-Americans in non-treatment studies involving multiple blood draws and follow-up visits can be accomplished effectively. This paper provides valuable information for investigators embarking on non-treatment laboratory-based studies among minority populations.

## Competing interests

Dr. Ahluwalia is a consultant to Pfizer Inc.

## Authors' contributions

All authors have made substantial contributions to the intellectual content of the manuscript as follows: BF, CAB and IO participated in the acquisition of data; BF, LSC and CAB participated in drafting the manuscript; all authors participated in the concept and design of the study, interpretation of data, review and approval of the final version of the manuscript.

## Pre-publication history

The pre-publication history for this paper can be accessed here:

http://www.biomedcentral.com/1471-2288/10/6/prepub

## References

[B1] USDHHS Healthy people 2000: National health promotion and disease prevention objectives full report with commentary 1991 Washington, D.C.: Government Printing Office

[B2] CDC Health Disparities Experienced by Black or African-Americans - United States MMWR 2005 54 01 1 3 15647722

[B3] Roberson NL Clinical trial participation: Viewpoints from racial/ethnic groups Cancer 1994 74 Suppl 9 2687 2691 10.1002/1097-0142(19941101)74:9+<2687::AID-CNCR2820741817>3.0.CO;2-B 7954287

[B4] Holcombe RF Jacobson J Li A Moinpour CM Inclusion of black Americans in oncology clinical trials: the Louisiana State University Medical Center experience Am J Clin Oncol 1999 22 1 18 21 10.1097/00000421-199902000-00005 10025373

[B5] Swanson GM Ward AJ Recruiting minorities into clinical trials: toward a participant-friendly system J Natl Cancer Inst 1995 87 23 1747 1759 10.1093/jnci/87.23.1747 7473831

[B6] Dennis BP Neese JB Recruitment and retention of African American elders into community-based research: lessons learned Arch Psychiatr Nurs 2000 14 1 3 11 10.1016/S0883-9417(00)80003-5 10692801

[B7] Evelyn B Toigo T Banks D Pohl D Gray K Robins B Ernat J Participation of racial/ethnic groups in clinical trials and race-related labeling: a review of new molecular entities approved 1995-1999 J Natl Med Assoc 2001 93 Suppl 12 S18 S24 PMC2719997 11798060

[B8] Ganz PA Clinical trials. Concerns of the patient and the public Cancer 1990 65 Suppl 10 2394 2399 10.1002/1097-0142(19900515)65:10+<2394::AID-CNCR2820651509>3.0.CO;2-# 2185874

[B9] Giuliano AR Mokuau N Hughes C Tortolero-Luna G Risendal B Ho RCS Prewitt TE McCaskill-Stevens WJ Participation of minorities in cancer research: the influence of structural, cultural, and linguistic factors Ann Epidemiol 2000 10 Suppl 8 S22 S34 10.1016/S1047-2797(00)00195-2 11189089

[B10] King TE Jr Racial disparities in clinical trials N Engl J Med 2002 346 18 1400 1402 10.1056/NEJM200205023461812 11986416

[B11] Powell JH Fleming Y Making medicines for America: the case for clinical trial diversity J Natl Med Assoc 2000 92 11 507 514 11152082 PMC2568328

[B12] Shavers-Hornaday VL Lynch CF Burmeister LF Torner JC Why are African Americans under-represented in medical research studies? Impediments to participation Ethn Health 1997 2 1-2 31 45 9395587 10.1080/13557858.1997.9961813

[B13] Stark N Paskett E Bell R Cooper MR Walker E Wilson A Tatum C Increasing participation of minorities in cancer clinical trials: summary of the "Moving Beyond the Barriers" Conference in North Carolina J Natl Med Assoc 2002 94 1 31 39 11837350 PMC2594090

[B14] Svensson CK Representation of American blacks in clinical trials of new drugs JAMA 1989 261 2 263 265 10.1001/jama.261.2.263 2909024

[B15] El-Sadr W Capps L The challenge of minority recruitment in clinical trials for AIDS JAMA 1992 267 7 954 957 10.1001/jama.267.7.954 1734108

[B16] Moyé LA Powell JH Evaluation of ethnic minorities and gender effects in clinical trials: opportunities lost and rediscovered J Natl Med Assoc 2001 93 Suppl 12 29S 34S 11798062 PMC2719996

[B17] Nicholson WK Brown AF Gathe J Grumbach K Washington AE Pérez-Stable EJ Hormone replacement therapy for African American women: missed opportunities for effective intervention Menopause 1999 6 2 147 155 10.1097/00042192-199906020-00012 10374222

[B18] D'Agostino RB Sr Grundy S Sullivan LM Wilson P CHD Risk Prediction Group Validation of the Framingham coronary heart disease prediction scores: results of a multiple ethnic groups investigation JAMA 2001 286 2 180 187 10.1001/jama.286.2.180 11448281

[B19] Bonner GJ Miles TP Participation of African Americans in clinical research Neuroepidemiology 1997 16 6 281 284 10.1159/000109698 9430127

[B20] Gorelick PB Harris Y Burnett B Bonecutter FJ The recruitment triangle: reasons why African Americans enroll refuse to enroll, or voluntarily withdraw from a clinical trial J Natl Med Assoc 1998 90 3 141 145 9549977 PMC2608331

[B21] Paskett ED DeGraffinreid C Tatum CM Margitić SE The recruitment of African Americans to cancer prevention and control studies Prev Med 1996 25 5 547 553 10.1006/pmed.1996.0088 8888322

[B22] Escobar-Chaves SL Tortolero SR Mâsse LC Watson KB Fulton JE Recruiting and retaining minority women: findings from the Women on the Move study Ethn Dis 2002 12 2 242 251 12019934

[B23] Corbie-Smith G Thomas SB Williams MV Moody-Ayers S Attitudes and beliefs of African Americans toward participation in medical research J Gen Intern Med 1999 14 9 537 546 10.1046/j.1525-1497.1999.07048.x 10491242 PMC1496744

[B24] Corbie-Smith G Thomas SB St George DM Distrust, race, and research Arch Intern Med 2002 162 21 2458 2463 10.1001/archinte.162.21.2458 12437405

[B25] Mouton CP Harris S Rovi S Solorzano P Johnson MS Barriers to black women's participation in cancer clinical trials J Natl Med Assoc 1997 89 11 721 727 9375475 PMC2608280

[B26] Stoy DB Curtis RC Dameworth KS Dowdy AA Hegland J Levin JA Sousoulas BG The successful recruitment of elderly black subjects in a clinical trial: the CRISP experience. Cholesterol Reduction in Seniors Program J Natl Med Assoc 1995 87 4 280 287 7752281 PMC2607802

[B27] Harris RE Zang EA Anderson JI Wynder EL Race and sex differences in lung cancer risk associated with cigarette smoking Int J Epidemiol 1993 22 4 592 599 10.1093/ije/22.4.592 8225730

[B28] Okuyemi K Cox L Choi WS Ahluwalia JS Isaacs SL Smoking cessation in U.S. ethnic minority populations VA in the Vanguard: Building on Success in Smoking Cessation 2005

[B29] Orleans CT Schoenbach VJ Salmon MA Strecher VJ Kalsbeek W Quade D Brooks EF Konrad TR Blackmon C Watts CD A survey of smoking and quitting patterns among black Americans Am J Public Health 1989 79 2 176 181 10.2105/AJPH.79.2.176 2913836 PMC1349929

[B30] Sidney S Tekawa I Friedman GD Mentholated cigarette use among multiphasic examinees 1979-86 Am J Public Health 1989 79 10 1415 6 10.2105/AJPH.79.10.1415 2782516 PMC1350190

[B31] Cummings KM Giovino G Mendicino AJ Cigarette advertising and black-white differences in brand preference Public Health Rep 1987 102 6 698 701 3120235 PMC1477979

[B32] Okuyemi KS Ahluwalia JS Ebersole-Robinson M Catley D Mayo MS Resnicow K Does menthol attenuate the effect of bupropion among African American smokers? Addiction 2003 98 10 1387 1393 10.1046/j.1360-0443.2003.00443.x 14519175

[B33] Okuyemi KS Faseru B Sanderson Cox L Bronars CA Ahluwalia JS Relationship between menthol cigarettes and smoking cessation among African American light smokers Addiction 2007 102 12 1979 1986 10.1111/j.1360-0443.2007.02010.x 17916223

[B34] Okuyemi KS Cox LS Nollen NL Snow TM Kaur H Choi W Nazir N Mayo MS Ahluwalia JS Baseline characteristics and recruitment strategies in a randomized clinical trial of African-American light smokers Am J Health Promot 2007 21 3 183 191 17233236 10.4278/0890-1171-21.3.183

[B35] Cox LS Cupertino AP Mussulman LM Nazir N Greiner KA Mahnken JD Ahluwalia JS Ellerbeck EF Design and baseline characteristics from the KAN-QUIT disease management intervention for rural smokers in primary care Prev Med 2008 47 2 200 205 10.1016/j.ypmed.2008.04.013 18544464 PMC2577567

[B36] Cupertino PA Richter KP Cox LS Nazir N Greiner AK Ahluwalia JS Ellerbeck EF Smoking cessation pharmacotherapy preferences in rural primary care Nicotine Tob Res 2008 10 2 301 307 10.1080/14622200701825817 18236294 PMC2821185

[B37] Harris KJ Ahluwalia JS Catley D Okuyemi KS Mayo MS Resnikow K Successful recruitment of minorities into clinical trials: The Kick It at Swope project Nicotine Tob Res 2003 5 4 575 84 10.1080/1462220031000118540 12959796

[B38] Ahluwalia JS Resnicow K Clark WS Knowledge about smoking reasons for smoking, and reasons for wishing to quit in inner-city African Americans Ethn Dis 1998 8 3 385 393 9926909

[B39] Ahluwalia JS McNagny SE Clark WS Smoking cessation among inner-city African Americans using the nicotine transdermal patch J Gen Intern Med 1998 13 1 1 8 10.1046/j.1525-1497.1998.00001.x 9462488 PMC1496888

[B40] Ahluwalia JS Richter K Mayo MS Ahluwalia HK Choi WS Schmelzle KH Resnicow K African American smokers interested and eligible for a smoking cessation clinical trial: predictors of not returning for randomization Ann Epidemiol 2002 12 3 206 212 10.1016/S1047-2797(01)00305-2 11897179

[B41] Woods MN Harris KJ Mayo MS Catley D Scheibmeir M Ahluwalia JS Participation of African Americans in a smoking cessation trial: a quantitative and qualitative study J Natl Med Assoc 2002 94 7 609 618 12126287 PMC2594312

[B42] Ahluwalia JS Harris KJ Catley D Okuyemi KS Mayo MS Sustained-release bupropion for smoking cessation in African Americans: a randomized controlled trial JAMA 2002 288 4 468 474 10.1001/jama.288.4.468 12132977

[B43] Okuyemi KS Richter KP Ahluwalia JS Mosier MC Nazir N Resnicow K Smoking reduction practices among African American smokers Nicotine Tob Res 2002 4 Suppl 2 S167 173 10.1080/1462220021000032744 12573177

[B44] Okuyemi KS Scheibmeir M Butler J Ahluwalia JS Perceptions of smoking among African American light smokers Subst Abus 2003 24 3 191 193 12913368 10.1080/08897070309511548

[B45] Okah FA Okuyemi KS McCarter KS Harris KJ Catley D Kaur H Ahluwalia JS Predicting adoption of home smoking restriction by inner-city black smokers Arch Pediatr Adolesc Med 2003 157 12 1202 1205 10.1001/archpedi.157.12.1202 14662576

[B46] Okuyemi KS Ahluwalia JS Banks R Harris KJ Mosier MC Nazir N Powell J Differences in smoking and quitting experiences by levels of smoking among African Americans Ethn Dis 2004 14 1 127 133 15002932

[B47] Harris KJ Okuyemi KS Catley D Mayo MS Ge B Ahluwalia JS Predictors of smoking cessation among African-Americans enrolled in a randomized controlled trial of bupropion Prev Med 2004 38 4 498 502 10.1016/j.ypmed.2003.12.008 15020185

[B48] Nollen NL Catley D Davies G Hall M Ahluwalia JS Religiosity, social support, and smoking cessation among urban African American smokers Addict Behav 2005 30 6 1225 1229 10.1016/j.addbeh.2004.10.004 15925130

[B49] Okuyemi KS Faseru B Sanderson Cox L Bronars CA Ahluwalia JS Relationship between menthol cigarettes and smoking cessation among African American light smokers Addiction 2007 102 12 1979 1986 10.1111/j.1360-0443.2007.02010.x 17916223

[B50] Cox LS Bronars CA Thomas JL Okuyemi KS King G Mayo MS Ahluwalia JS Achieving high rates of consent for genetic testing among African American smokers Nicotine Tob Res 2007 9 6 711 716 10.1080/14622200701365228 17558828

[B51] Okuyemi KS Powell JN Savage CR Hall SB Nollen N Holsen LM McClernon FJ Ahluwalia JS Enhanced cue-elicited brain activation in African American compared with Caucasian smokers: an fMRI study Addict Biol 2006 11 1 97 106 10.1111/j.1369-1600.2006.00007.x 16759342

[B52] Ahluwalia JS Okuyemi K Nollen N Choi WS Kaur H Pulvers K Mayo MS The effects of nicotine gum and counseling among African American light smokers: a 2 × 2 factorial design Addiction 2006 101 6 883 891 10.1111/j.1360-0443.2006.01461.x 16696632

[B53] Woods MN Harris KJ Ahluwalia JS Schmelzle KH Mayo MS Smoking in urban African Americans: behaviors, gender differences and motivation to quit Ethn Dis 2001 11 3 532 539 11572418

[B54] Nollen NL Mayo MS Sanderson Cox L Okuyemi KS Choi WS Kaur H Ahluwalia JS Predictors of quitting among African American light smokers enrolled in a randomized, placebo-controlled trial J Gen Intern Med 2006 21 6 590 595 10.1111/j.1525-1497.2006.00404.x 16808741 PMC1924642

[B55] Brooks D Charleston J Dowie D Gabriel A Hall YB Hiremath L Lightfoot T Sika M Smith WC Wang X Predictors of participant adherence and retention in the African American Study of Kidney Disease and Hypertension Nephrol Nurs J 2008 35 2 133 42 18472682

[B56] Kabat GC Hebert JR Use of mentholated cigarettes and lung cancer risk Cancer Res 1991 51 24 6510 3 1742723

